# Exploring causal associations between interleukins, their receptors, and acute respiratory distress syndrome: a bidirectional-Mendelian Randomization study

**DOI:** 10.3389/fgene.2024.1447661

**Published:** 2024-11-21

**Authors:** Jia-wei Ma, Ji-chun Tang, Hong-zhi Fang

**Affiliations:** ^1^ Department of Critical Care Medicine, Wuxi No.2 People’s Hospital, Jiangnan University Medical Center, Wuxi, China; ^2^ Department of Critical Care Medicine, Aheqi County People’s Hospital, Aheqi, Xinjiang, China; ^3^ Department of Anesthesiology, Affiliated Hospital of Jiangnan University, Wuxi, China; ^4^ Emergency Department, Wuxi No.2 People’s Hospital, Jiangnan University Medical Center, Wuxi, China

**Keywords:** acute respiratory distress syndrome, causal associations, genetic epidemiology, interleukins, mendelian randomization

## Abstract

**Background:**

Acute Respiratory Distress Syndrome (ARDS) is a severe lung condition posing significant health risks. Observational studies have indicated a potential linkage between ARDS and various interleukins, such as Interleukin-8, −2, −6, and −1β. However, the causal relationships between specific interleukins, their receptors, and ARDS remain unclear.

**Methods:**

This study employed bidirectional Mendelian Randomization (MR) to investigate the causal associations between 197 different interleukins, interleukin receptors, and ARDS. GWAS summary data for interleukins and their receptors were sourced from publicly available studies, while ARDS data were derived from the FinnGen database. Wald Ratio and Inverse Variance Weighted (IVW) methods were primarily utilized in the MR analyses. Reverse MR was also conducted to explore reverse causations.

**Results:**

The study identified significant causal relationships between several interleukins, their receptors and ARDS. Specifically, Interleukin-17 receptor D (IL-17RD) (OR = 0.67, 95%CI = 0.51–0.89, *p* = 0.006), Interleukin-22 receptor subunit alpha-1 (IL22RA-1) (OR = 1.45, 95%CI = 1.08–1.96, *p* = 0.014), Interleukin-20 receptor subunit alpha (IL20RA) (OR = 0.53, 95%CI = 0.32–0.89, *p* = 0.016), Interleukin-22 (IL-22) (OR = 3.60, 95%CI = 1.20–10.83, *p* = 0.022), Interleukin-23 receptor (IL-23R) (OR = 2.14, 95%CI = 1.10–4.17, *p* = 0.025), Single Ig IL-1-related receptor (SIGIRR) (OR = 1.22, 95%CI = 1.00–1.48, *p* = 0.047) showed notable associations with ARDS. No reverse causal relationships were found on results above, and neither heterogeneity nor pleiotropy was detected in the analysis.

**Conclusion:**

This study elucidates the causal connections between specific interleukins, their receptors and ARDS, contributing significantly to the understanding of ARDS pathogenesis. These findings offer a foundation for further research and potential therapeutic interventions targeting these interleukins and receptors in ARDS management. The absence of reverse causation and pleiotropy, heterogeneity reinforces the robustness of these associations.

## Introduction

Acute respiratory distress syndrome (ARDS) is a critically severe lung condition that represents a major health challenge globally (Ervin et al., 2020; Ganeriwal et al., 2023; Hussain et al., 2021; Jayasimhan, Foster, Chang and Hancox, 2021; Wang et al., 2019). Characterized by rapid onset of widespread inflammation in the lungs, ARDS leads to the accumulation of fluid in the alveoli, which are tiny air sacs responsible for gas exchange (McKelvey et al., 2022; Sivapalan, Bonnesen and Jensen, 2020). This accumulation severely impairs oxygenation, resulting in life-threatening levels of low blood oxygen and high carbon dioxide. ARDS can be triggered by a variety of factors, including severe infections like pneumonia, trauma, sepsis, or inhalation of harmful substances. The syndrome is a leading cause of mortality and morbidity in intensive care units, with a mortality rate ranging from 35% to 50%. Despite advancements in supportive care, the lack of specific therapies targeting the underlying pathophysiology of ARDS continues to be a significant hurdle (Loh, Gan, Ong, Wong and Lee, 2020). The condition not only poses an immediate threat to life but also leads to long-term complications for survivors, including persistent lung damage and reduced quality of life due to physical and psychological burdens. As such, understanding the pathogenic mechanisms of ARDS is crucial for developing targeted treatments and improving patient outcomes. This urgency is further underscored by recent global health events, such as the COVID-19 pandemic, which saw a surge in ARDS cases, highlighting its relevance in contemporary medical and public health spheres.

The relationship between ARDS and interleukins (ILs), along with their receptors, is an area of keen interest and ongoing research. Interleukins are a group of cytokines, which are signaling molecules that play a pivotal role in the immune system. They are known for their involvement in inflammatory and immune responses, making them crucial players in conditions like ARDS (Saki et al., 2023). Observational studies have increasingly highlighted a significant association between ARDS and various interleukins, such as Interleukin-8 (IL-8), Interleukin-2 (IL-2), Interleukin-6 (IL-6), and Interleukin-1β (IL-1β) (Wang et al., 2019). These interleukins are thought to contribute to the pathogenesis of ARDS through different mechanisms (Loinjak et al., 2023; Wiche Salinas, Zheng, Routy and Ancuta, 2020). For instance, IL-8, known for its role in chemotaxis, actively recruits neutrophils to the site of inflammation, potentially exacerbating lung injury in ARDS. Similarly, IL-6, a pro-inflammatory cytokine, is implicated in the systemic inflammatory response that characterizes ARDS, promoting further lung damage. IL-2 plays a dual role in immune regulation and has been associated with both the exacerbation and resolution of inflammatory responses in ARDS, suggesting a complex interplay in its functions. IL-1β is another critical pro-inflammatory cytokine, believed to amplify the inflammatory cascade in ARDS. Additionally, the receptors for these interleukins are equally significant, as they are the mediators through which interleukins exert their effects. The interaction between interleukins and their receptors can trigger a cascade of intracellular signaling pathways, leading to various inflammatory responses. This interaction is particularly pertinent in the lungs, where an exaggerated response can result in the severe pulmonary edema characteristic of ARDS. The intricate relationship between these interleukins and their receptors in the context of ARDS points towards a complex network of inflammatory signaling that may hold the key to understanding the pathophysiology of ARDS. Thus, these cytokines and their interactions not only provide insight into the disease mechanism but also present potential therapeutic targets for managing and treating ARDS. This is especially critical considering the current limitations in ARDS treatment, which primarily focuses on supportive care rather than targeting specific pathogenic pathways. Understanding the roles of interleukins and their receptors in ARDS could lead to more effective, targeted therapies, potentially improving outcomes for patients suffering from this severe condition.

Mendelian Randomization (MR) is a research method that leverages genetic variations to study causal relationships between potentially modifiable risk factors and health outcomes (Emdin et al., 2017; Hartwig et al., 2018; Sanderson et al., 2022; Weith and Beyer, 2023). This approach is based on the principle of Mendel’s laws of inheritance, where genetic variants are randomly assorted at conception, much like the random allocation in a clinical trial. This random assortment helps to mitigate confounding factors and reverse causation, common issues in observational studies, thus providing a more robust inference of causality. In our study, we employed bidirectional Mendelian Randomization to unravel the causal associations between interleukins (ILs), their receptors, and ARDS. The bidirectional aspect of our MR approach meant we not only investigated the potential causal impact of interleukins and their receptors on the risk of developing ARDS, but also explored whether ARDS could influence the levels or functioning of these interleukins and receptors. This comprehensive approach provides a more nuanced understanding of the relationship between these entities. To implement this method, we utilized Genome-Wide Association Studies (GWAS) summary data for interleukins and their receptors from publicly available databases. This data provided us with a plethora of genetic variants associated with the levels or functioning of these interleukins and receptors. For ARDS data, we tapped into the FinnGen database, which is a rich resource of genetic and health information from Finnish populations. Our primary analytical tools were the Wald Ratio and Inverse Variance Weighted (IVW) methods, which are well-established techniques in MR analyses for estimating causal effects. The Wald Ratio method uses a single genetic variant as an instrumental variable, while IVW uses multiple variants, thereby enhancing the reliability and validity of our findings. By applying these robust statistical methods, our study not only contributes to the understanding of ARDS pathogenesis but also opens up new avenues for potential therapeutic interventions targeting specific interleukins and receptors in the management of ARDS.

## Materials and methods

### Data source

Our study utilized Genome-Wide Association Studies (GWAS) summary data of interleukins and their receptors from publicly accessible GWAS research databases, specifically the MRC Integrative Epidemiology Unit GWAS database (https://gwas.mrcieu.ac.uk/). Our analysis encompassed data from seven distinct studies, incorporating a total of 197 GWAS entries related to interleukins and their receptors (see detailed information in Supplementary Table S1). This comprehensive dataset included notable contributions from several researchers. Ari V Ahola-Olli and colleagues conducted a GWAS in a Finnish cohort of up to 8,293 individuals, identifying 27 genome-wide significant loci (*p* < 1.2 × 10^−9^) for various cytokines, with 15 variants showing expression quantitative trait loci in whole blood (Ahola-Olli et al., 2017). Suhre et al. (2017) described a GWAS employing a multiplexed aptamer-based affinity proteomics platform, quantifying 539 associations (protein quantitative trait loci or pQTLs) between protein levels and gene variants in a German cohort, with over half of these associations replicated in Arab and Asian cohorts. Furthermore, Lasse Folkersen and colleagues reported GWAS results for 83 proteins related to cardiovascular disease (CVD) in 3,394 individuals with multiple CVD risk factors, identifying 79 loci for 83 plasma protein biomarkers in cardiovascular disease (Ripatti et al., 2017). Sun et al. (2018) work characterized the genetic architecture of the human plasma proteome in healthy blood donors from the INTERVAL study, uncovering 1,927 genetic associations with 1,478 proteins, including trans associations for 1,104 proteins. Steven Offenbacher’s study reported a GWAS for high gingival crevicular fluid IL-1β expression among 4,910 European-American adults, identifying association signals in the IL37 locus (Offenbacher et al., 2018). Robert F Hillary performed genome- and epigenome-wide association studies (GWAS/EWAS) on 70 plasma-derived inflammatory protein biomarkers in healthy older adults (Lothian Birth Cohort 1936; n = 876; using the Olink^®^ inflammation panel), employing a Bayesian framework (BayesR+) (Hillary et al., 2020). This approach accounted for issues related to data structure and unknown confounding variables, with sensitivity analyses using ordinary least squares (OLS) and mixed model-based approaches, identifying 13 SNPs associated with 13 proteins. Finally, Arthur Gilly and colleagues comprehensively assessed the genetic architecture of 257 circulating protein biomarkers of cardiometabolic relevance through high-depth whole-genome sequencing (WGS) in 1,328 individuals (Gilly et al., 2020). This study discovered 131 independent sequence variant associations (*p* < 7.45 × 10^−11^) across the allele frequency spectrum, all of which replicated in an independent cohort (n = 1,605), and identified for the first time replicating evidence for rare-variant cis-acting protein quantitative trait loci for five genes, involving both coding and noncoding variation.

The Genome-Wide Association Studies (GWAS) summary data utilized in this research were sourced from the FinnGen project (specifically from the dataset J10_ARDS, comprising 328 cases and 372,077 controls, accessible at https://r10.risteys.finngen.fi/endpoints/J10_ARDS#dialog-view-original-rules). The endpoint definition for ARDS (J10_ARDS) in the FinnGen study begins with 429,209 individuals. No sex-specific rules or condition checks were applied. The key criteria include ICD-10 (J80) or ICD-9 (5,185) codes for hospital discharge and cause of death. The data were derived from 2 out of 7 registries. Individuals were then filtered based on genotype quality control (QC), resulting in 375 individuals meeting the endpoint definition. The ARDS endpoint is excluded from the J10_INTERSTITIUM control group, with the condition being at level 3 in the ICD hierarchy and first used in the DF6 data freeze. FinnGen represents an ambitious public-private partnership, initiated with the objective of collecting and analyzing genomic and health data from approximately 500,000 participants in Finnish biobanks. FinnGen not only serves as a beacon for large-scale genomic research but also as a testament to the power of cooperative engagement across various sectors and communities.

### Instrumental variants selection

**SCHEME 1:**
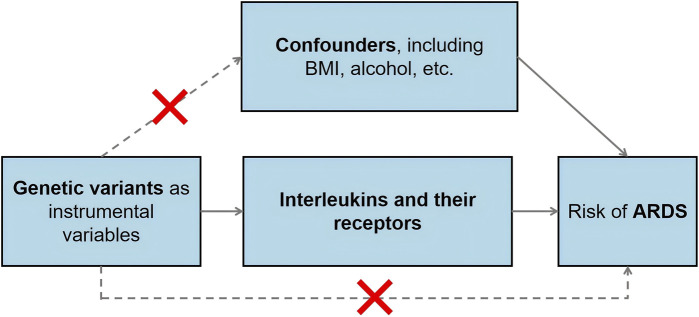
The schematic diagram illustrating the MR study design.

In our study, we identified genetic variants associated with various interleukins and their receptors, drawing from the comprehensive GWAS dataset previously outlined. As shown in Scheme 1, the selection of instrumental variables, crucial for Mendelian Randomization analysis, was meticulously conducted with adherence to stringent criteria tailored to each dataset. Our selection criteria for single nucleotide polymorphisms (SNPs) were rigorously defined. We established a significance threshold at *p* < 5 × 10^−8^, coupled with a stringent linkage disequilibrium cutoff (*R* ^2^ < 0.001 within a 10,000 kb window). This approach was employed to ensure robust analytical power and to minimize the potential for confounding due to correlated genetic variants. For datasets where the application of the *p* < 5 × 10^−8^ threshold did not yield instrumental variables, we adopted a more lenient threshold of *p* < 5 × 10^−6^. This step was crucial for extracting instrumental variables from these specific datasets while still maintaining a level of rigor in our selection process. Additionally, we excluded palindromic SNPs from our analysis. This exclusion was a precautionary measure to prevent any potential measurement bias that could arise due to ambiguity in strand alignment. Palindromic SNPs, due to their symmetrical nucleotide composition, can pose challenges in accurate genotyping, hence their exclusion was deemed necessary to enhance the validity of our findings. Overall, these stringent selection procedures for genetic variants were pivotal in bolstering the reliability of our Mendelian Randomization analysis, ensuring that the instrumental variables used were both relevant and robust, thereby contributing to the credibility of our study’s conclusions.

### Mendelian Randomization

**SCHEME 2:**
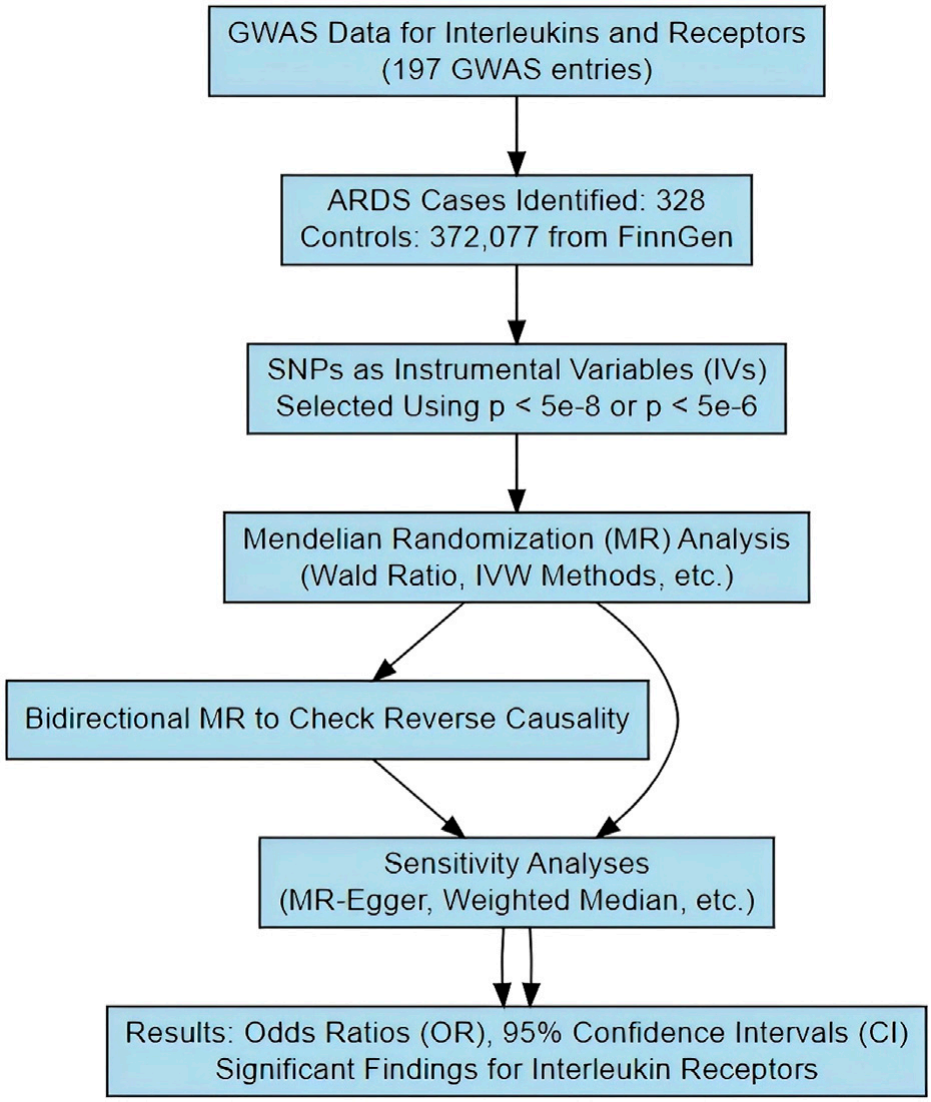
The schematic diagram illustrating the study process.

As shown in Scheme 2, we implemented the Wald ratio and the inverse-variance weighted (IVW) method as the primary analytical tools for Mendelian Randomization (MR) analysis. The Wald ratio was employed in scenarios where only a single instrumental variable was available, while the IVW method was utilized when two or more instrumental variables were present. These methods are well-established in the field of genetic epidemiology and are instrumental in elucidating causal relationships between genetic variants and phenotypic traits. For traits that demonstrated significant findings via the Wald ratio or IVW method, we conducted comprehensive sensitivity analyses using a suite of alternative MR methods. These included the weighted median estimator, MR-Egger regression, simple mode, and weighted mode. Such analyses were crucial in affirming the robustness and consistency of our results. The findings were quantified and presented as odds ratios (ORs) with corresponding 95% confidence intervals (CIs), providing a measure of the strength and precision of the identified associations. To ensure the validity of the MR assumptions in our analysis, we rigorously tested for the absence of pleiotropy and heterogeneity. This was accomplished using the Cochrane Q test for heterogeneity and the MR-Egger intercept regression for pleiotropy. Additionally, a leave-one-out analysis was conducted to reinforce the robustness of our MR findings. Given the extensive scope of our study, which analyzed a total of 197 lipidomic and amino acid traits, we set a Bonferroni-adjusted *p* threshold of 0.0002 (calculated as 0.05/197) to determine significant associations. However, acknowledging the potential interdependencies among these exposures, we recognized that the Bonferroni correction might impose an overly stringent criterion. Therefore, we also applied a more lenient threshold of *p* < 0.01 for correcting multiple testing results as nominally significant (Zhu et al., 2024). In instances where potential causal relationships were indicated (*p* < 0.05), we employed reverse Mendelian Randomization to investigate the reverse causation between interleukins, their receptors, and ARDS. The selection of instrumental variables and the choice of methods for this reverse analysis were consistent with the approach described for the forward MR analysis. This bidirectional exploration provided a more comprehensive understanding of the dynamics between genetic variants and the phenotypic traits under study.

### Statistical analysis

The methodology and design of this study strictly adhere to the STROBE-MR checklist (Skrivankova et al., 2021). All statistical analyses were meticulously performed using the R software (Version 4.1.3).

## Results

**FIGURE 1:**
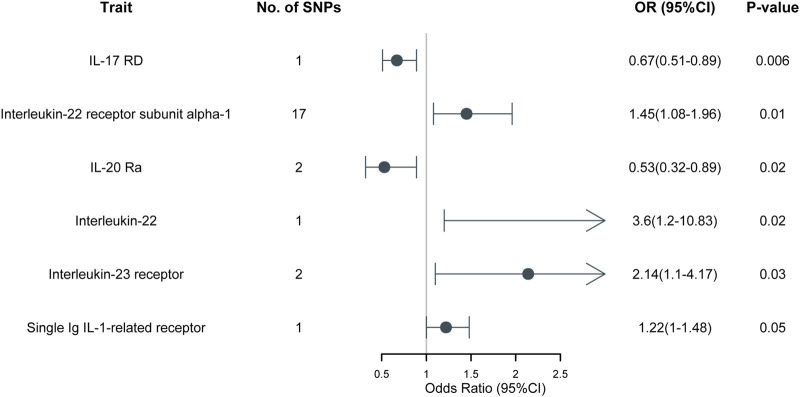
MR results of 197 different interleukins, interleukin receptors on ARDS.

**FIGURE 2:**
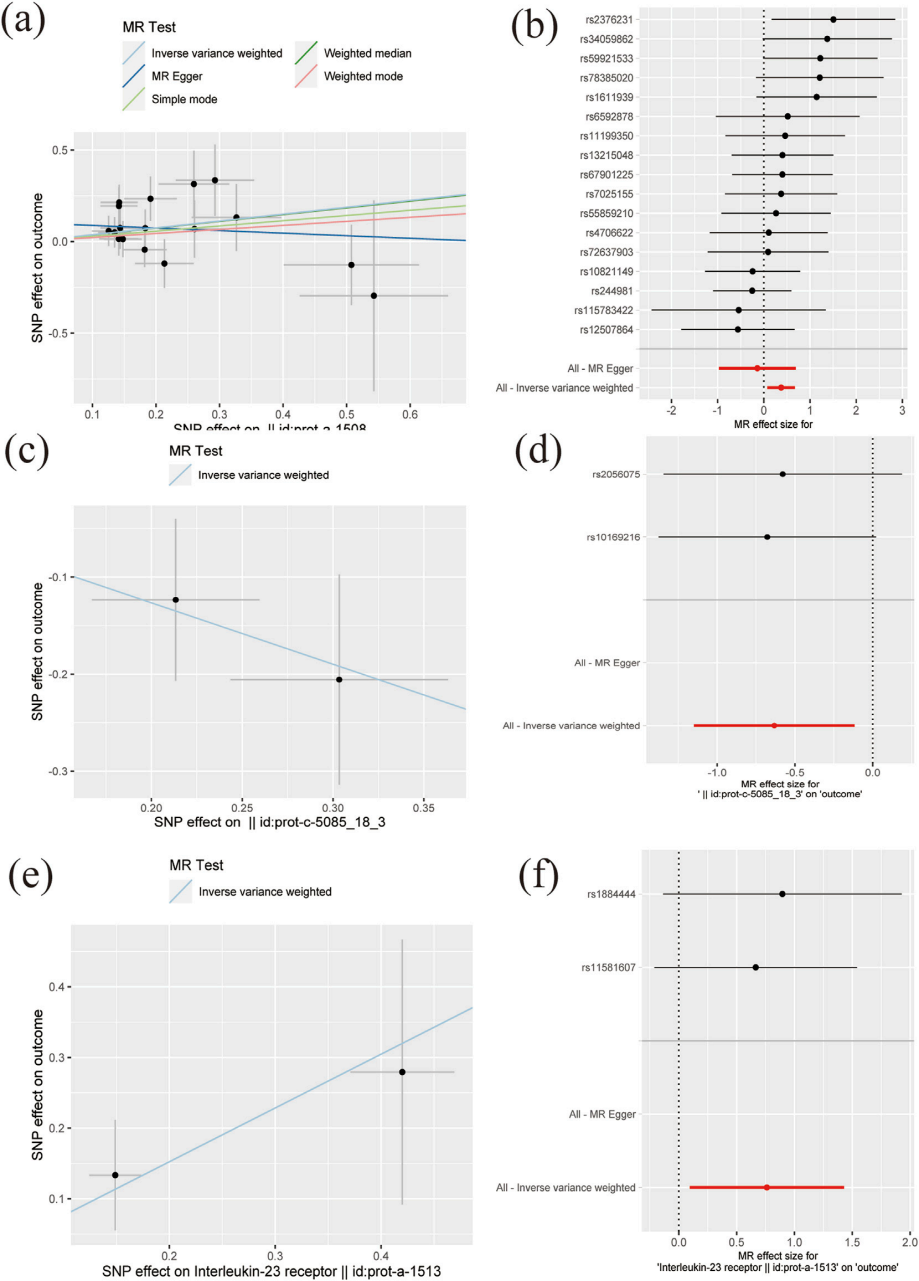
The scatter plots and forest plots of MR results **(A–B)** Interleukin-22 receptor subunit alpha-1 **(C–D)** IL-20 Ra **(E–F)** Interleukin-23 receptor.

**FIGURE 3:**
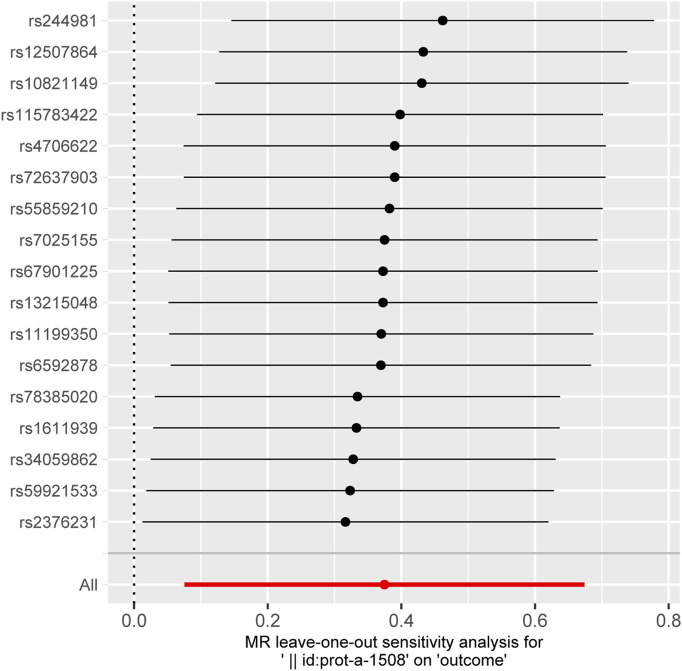
The plot of leave-one-out sensitivity analysis for Interleukin-22 receptor subunit alpha-1.

All the IVs for 197 different interleukins, interleukin receptors were listed in Supplementary Table S2 with *p*_threshold_ = 5 × 10^−8^ or 5 × 10^−6^. This table includes information including the SNP rsid, chromosomal location, effect sizes (betas), standard errors (SEs), and *p*-values for both the exposure and outcome variables. Comprehensive results of all MR analysis are presented in Supplementary Table S3. As shown in Figure 1, for a threshold of *p* = 0.05, we found that those interleukins, interleukin receptors have association with ARDS (Interleukin-17 receptor D (IL-17RD) (OR = 0.67, 95%CI = 0.51–0.89, *p* = 0.006), Interleukin-22 receptor subunit alpha-1 (IL22RA-1) (OR = 1.45, 95%CI = 1.08–1.96, *p* = 0.014), Interleukin-20 receptor subunit alpha (IL20RA) (OR = 0.53, 95%CI = 0.32–0.89, *p* = 0.016), Interleukin-22 (IL-22) (OR = 3.60, 95%CI = 1.20–10.83, *p* = 0.022), Interleukin-23 receptor (IL-23R) (OR = 2.14, 95%CI = 1.10–4.17, *p* = 0.025), Single Ig IL-1-related receptor (SIGIRR) (OR = 1.22, 95%CI = 1.00–1.48, *p* = 0.047)). However, upon applying the stringent Bonferroni correction, none of these associations retained their significance under the adjusted threshold. Notably, when a more lenient threshold of *p* = 0.01 was used, Interleukin-17 receptor D (IL-17RD) emerged as significantly associated (OR = 0.67, 95%CI = 0.51–0.89, *p* = 0.006), suggesting a potential protective effect. To assess the robustness of our findings, tests for heterogeneity and pleiotropy were conducted for interleukins and their receptors that showed potential associations with ARDS. No significant issues were identified, as shown in Supplementary Tables S4, S5. Furthermore, the MR-Egger intercept regression analysis did not reveal any evidence of horizontal pleiotropy (*p >* 0.05 for interleukins and their receptors with potential associations with ARDS) (Supplementary Table S4). Additionally, the Cochrane Q test did not detect heterogeneity in the study outcomes (all *p >* 0.05 for interleukins and their receptors with potential associations with ARDS) (Supplementary Table S5). Our analysis was further substantiated by a leave-one-out analysis, the results of which are presented in Figures 2, 3.

In an effort to explore reverse causality for the 6 MR results with *p <* 0.05, a reverse Mendelian randomization analysis was conducted, encompassing Interleukin-17 receptor D (IL-17RD), Interleukin-22 receptor subunit alpha-1 (IL22RA-1), Interleukin-20 receptor subunit alpha (IL20RA), Interleukin-22 (IL-22), Interleukin-23 receptor (IL-23R), and Single Ig IL-1-related receptor (SIGIRR). All IVs for ARDS (with a *p* threshold of 5 × 10^−6^) and their respective effect sizes (betas), standard errors (SEs), and *p* for the six traits were meticulously documented in Supplementary Table S6. The comprehensive results of all reverse MR analyses are provided in Supplementary Table S7. Notably, no evidence of reverse causality was observed.

In conclusion, our study highlights associations between interleukins and interleukin receptors and ARDS, particularly for Interleukin-17 receptor D (IL-17RD) at a more lenient *p*-value threshold. These findings were found to be robust, with no significant issues of heterogeneity or pleiotropy detected. Reverse causality analyses did not reveal any causal relationships in the opposite direction.

## Discussion

In summary, this study aimed to elucidate the causal associations between a comprehensive set of 197 interleukins and interleukin receptors and ARDS using bidirectional Mendelian Randomization (MR) analysis. The investigation was prompted by previous observational studies that suggested potential links between specific interleukins and ARDS but lacked the ability to establish causality (Allen et al., 2007; Jorens et al., 1992; Kuzmanovic et al., 2024; Peukert et al., 2023). The results of our study revealed significant causal relationships between certain interleukins and ARDS. Notably, Interleukin-17 receptor D (IL-17RD), Interleukin-22 receptor subunit alpha-1 (IL22RA-1), Interleukin-20 receptor subunit alpha (IL20RA), Interleukin-22 (IL-22), Interleukin-23 receptor (IL-23R), and Single Ig IL-1-related receptor (SIGIRR) showed notable associations with ARDS. These findings provide valuable insights into the potential involvement of specific interleukins and their receptors in the pathogenesis of ARDS, shedding light on previously unexplored causal connections. Furthermore, the absence of reverse causal relationships, as demonstrated through reverse MR analysis, strengthens the evidence for a unidirectional causality from these interleukins and receptors to ARDS. This unidirectional causality hypothesis is further supported by the robustness of our findings, as indicated by the absence of heterogeneity and pleiotropy in the analysis. The stringent Bonferroni correction was applied to account for multiple testing, ensuring the reliability of the associations identified. These findings offer a foundation for further research aimed at understanding the mechanisms through which these interleukins and receptors may influence the development and progression of ARDS. Moreover, the potential therapeutic implications of targeting specific interleukins and receptors in ARDS management warrant exploration in future studies. Overall, this study advances our understanding of the complex interplay between interleukins, their receptors, and ARDS, providing valuable insights for both researchers and clinicians in the field of respiratory medicine.

The observed protective effect of Interleukin-17 receptor D (IL-17RD) against ARDS suggests intriguing potential mechanisms by which IL-17RD may influence ARDS susceptibility and pathogenesis. IL-17RD is known to be involved in the regulation of the immune response, particularly within the context of inflammation and host defense mechanisms (Billi and Gudjonsson, 2019; Girondel et al., 2020; Girondel and Meloche, 2021; Su et al., 2019). It is plausible that IL-17RD may modulate the immune and inflammatory responses in the lungs, thereby mitigating the severity of ARDS. One possible mechanism could involve the regulation of pro-inflammatory cytokines and chemokines. IL-17RD may exert a suppressive effect on the production or signaling of pro-inflammatory mediators, leading to a dampened inflammatory cascade in the lung tissue (Akhter et al., 2023). This, in turn, could reduce the extent of lung injury and edema, two hallmark features of ARDS (Girondel and Meloche, 2021; Pande et al., 2021). Furthermore, IL-17RD may play a role in preserving the integrity of the pulmonary endothelial and epithelial barriers. ARDS is characterized by increased vascular permeability and disruption of the alveolar-capillary membrane (Vassiliou et al., 2020). IL-17RD might contribute to the maintenance of barrier function, thus reducing leakage of fluids and proteins into the alveolar space (Ge et al., 2020). Another potential mechanism involves the regulation of immune cell recruitment and activation. IL-17RD may modulate the recruitment and activation of immune cells, such as neutrophils and macrophages, which are key contributors to the inflammatory response in ARDS (Song and Qian, 2013). By regulating the immune cell response, IL-17RD could potentially limit tissue damage and inflammation in the lungs (Girondel et al., 2020; Su et al., 2019). Overall, while the exact mechanisms underlying the protective effect of IL-17RD in ARDS warrant further investigation, it is plausible that its influence on immune regulation, inflammatory signaling, and pulmonary barrier integrity may collectively contribute to its observed beneficial role in mitigating ARDS susceptibility and severity. Further experimental studies are needed to elucidate the precise molecular pathways involved in IL-17RD-mediated protection against ARDS.

Beyond Interleukin-17 receptor D, Interleukin-22 receptor subunit alpha-1 showed a notable positive association with ARDS, indicating that it may contribute to the increased susceptibility to ARDS. Similarly, Interleukin-20 receptor subunit alpha was inversely associated with ARDS, suggesting a potential protective role against the development of ARDS. Additionally, Interleukin-22 demonstrated a strong association with ARDS, implying that this cytokine could play a critical role in the immune response leading to ARDS (Taghavi et al., 2021). Interleukin-23 receptor was also associated with ARDS, which supports the growing evidence of the involvement of the IL-23/IL-17 axis in inflammatory lung diseases (Ivanov et al., 2007). Lastly, the Single Ig IL-1-related receptor (SIGIRR) was marginally associated with ARDS. As a negative regulator of Toll-like and IL-1 receptor signaling, this finding could reflect SIGIRR’s modulatory role in controlling excessive inflammation during ARDS progression (Tian et al., 2023).

Mendelian Randomization offers several key advantages in this study. First, by leveraging genetic variants as instrumental variables, MR reduces the potential for confounding factors that often bias observational studies. This method provides a robust approach to assessing causal relationships between exposures, such as interleukins and their receptors, and outcomes like ARDS. Additionally, MR mitigates the issue of reverse causation, as genetic variants are fixed at conception, allowing for a more reliable inference of the directionality of the associations. These strengths make MR a powerful tool in uncovering potential causal links that are less susceptible to biases commonly found in traditional epidemiological approaches. However, it is important to acknowledge the limitations of this study. Firstly, our analysis relies on the assumptions of Mendelian Randomization, which assumes that the selected instrumental variables are valid and do not violate the exclusion restriction assumption. While we applied rigorous criteria for instrumental variable selection, the possibility of pleiotropic effects cannot be entirely ruled out, and there may be unmeasured confounders that could affect the results. Secondly, our study utilized data from publicly available GWAS databases, which may have inherent limitations, such as potential population stratification and limited diversity in study populations. These factors could introduce bias or affect the generalizability of our findings to broader populations. Furthermore, our study focused on genetic associations, and other non-genetic factors that could contribute to ARDS, such as environmental exposures or lifestyle factors, were not considered in this analysis. These factors could interact with genetic variants and influence ARDS risk. Lastly, the findings of this study provide evidence of association but do not establish causality definitively. Further experimental and clinical studies are necessary to validate the observed causal relationships and elucidate the underlying mechanisms.

## Conclusion

In conclusion, while this study contributes to our understanding of the potential causal associations between interleukins, their receptors, and ARDS, these findings should be interpreted in light of the aforementioned limitations. Future research should aim to address these limitations and provide a more comprehensive understanding of the complex interplay between interleukins and ARDS.

## Data Availability

The original contributions presented in the study are included in the article/Supplementary Material, further inquiries can be directed to the corresponding author.

## Abbreviations

ARDS, Acute Respiratory Distress Syndrome; GWAS, Genome-wide association study; IVW, Inverse Variance Weighted; MR, Mendelian randomization; SNPs, single nucleotide polymorphisms.
